# Association mapping integrated with image-based phenomics identifies SNPs and candidate genes underpinning fruit morphology and development in chile pepper (*Capsicum annum* L.)

**DOI:** 10.1093/g3journal/jkag116

**Published:** 2026-05-02

**Authors:** Ehtisham S Khokhar, Dennis N Lozada

**Affiliations:** Department of Plant and Environmental Sciences, New Mexico State University, Las Cruces, NM 88003, United States; Department of Plant and Environmental Sciences, New Mexico State University, Las Cruces, NM 88003, United States; Chile Pepper Institute, New Mexico State University, Las Cruces, NM 88003, United States

**Keywords:** image-based phenomics platform, marker–trait association analysis, fruit phenotypic diversity

## Abstract

Fruit morphology has a significant impact on the agronomic performance of chile peppers, influencing both yield potential and mechanical harvest efficiency. Through the integration of genome-wide association studies (GWAS) with Tomato Analyzer, an image-based phenomics tool, we aim to identify single nucleotide polymorphism (SNP) markers associated with fruit architecture and morphology. A Capsicum association mapping panel (CAMP) consisting of 129 genotypes was evaluated under an augmented field design for the 2024 growing season in New Mexico, United States. Best linear unbiased predictions (BLUPs) were reported for maximum fruit height (MAXH), maximum fruit width (MAXW), curved fruit height (CURH), width mid-height (WMH), height mid-width (HMW), area (ARA), perimeter (PER), 10-pod weight (PODW), and yield (YLD). Medium to low narrow-sense heritability (*h*^2^), ranging between 0.18 and 0.33, was observed. A medium to high Pearson correlation (*r* = 0.23 to 1.00) was observed for all traits except WMH. After filtration and imputation, 40,709 genotyping-by-sequencing (GBS) SNP markers were used to perform multilocus GWAS. A total of 169 SNP markers associated with seven basic fruit measurements across 12 chromosomes were identified. A SNP marker, *SCM002812.1_10016804* on chromosome 1, identified across multiple GWAS models was found to be associated with the potential candidate gene, *YABBY4*, which can regulate fruit development. Other candidate genes identified included *FRIGIDA*-like protein *3*, *COBRA*-like protein, and auxin-responsive protein *SAUR65* regulating flower, fruit, and shoot development. The findings of this study will be relevant for the development of molecular markers for marker-assisted selection, genomic selection for basic fruit morphology traits, and studying expression levels of genes regulating fruit development in chile peppers.

## Introduction

Chile peppers (*Capsicum* spp.) are one of the most important horticultural crops in the Solanaceae family, renowned for their economic significance, nutritional value, health benefits, and cultural association worldwide. Peppers originated in Western North America and South America and then dispersed to other parts of the world (Colonna et al. 2019). The genus *Capsicum* consists of approximately 36 species classified into 11 clades, among which *C. annuum*, *C. frutescens*, *C. chinense*, *C. baccatum*, and *C. pubescens* are domesticated (García et al. 2016). Among these cultivated species, *C. annuum* is widely adapted and grown for various fruit shapes, pod types, colors, pungency, growth habits, and maturity (Bosland and Votava 2012). In chile peppers, the pod type refers to a broad classification based on the general appearance of the fruit (e.g., jalapeño and New Mexican), whereas fruit shape is the precise description of the physical structure or geometry of a pepper fruit.

Selection for larger fruit with greater shape remains one of the major objectives for a chile pepper breeding program along with increased fruit mass, yield, varying levels of pungency, diverse colors and flavors, and compatibility with mechanical harvesting (Lozada et al. 2022; Khan et al. 2025; Khokhar et al. 2025). This also reflects consumer demand and preferences. The introgression of biological stress resistance traits, such as disease resistance against various pathogens, including *Phytophthora* spp., root-knot nematodes, and different types of viruses, also remained equally important (Changkwian et al. 2019; Jo et al. 2022; Sanogo et al. 2023). Wild relatives, such as the Mexican landrace pepper, Criollo de Morellos-334 (CM-334), have been extensively used in breeding for disease resistance; however, linkage drag associated with undesirable fruit morphology and quality remains a significant challenge. Adding to these challenges was the quantitative inheritance of fruit morphology, governed by multiple genes (Chunthawodtiporn et al. 2018). Wild relatives contribute valuable disease resistance genes; however, recovering desirable fruit morphology, quality, and yield from wide crosses requires multiple generations of selection. Employment of a multiomics strategy, including genomics, phenomics, transcriptomics, and metabolomics could dissect the complex genetic architecture of fruit morphology in chile peppers, enabling vegetable breeders to develop molecular markers for marker assisted breeding and genomic prediction of complex quantitative traits (Uffelmann et al. 2021; Lozada et al. 2022).

Genome-wide association studies (GWAS) are powerful tools for uncovering the genetic determinants of complex traits controlled by multiple loci, using marker–trait associations based on linkage disequilibrium (Cortes et al. 2021; Lozada et al. 2022). Unlike QTL mapping, which relies on biparental populations, GWAS utilizes diverse panels, thereby eliminating the need for structured crosses and enabling high genetic resolution due to historical recombination in diverse populations. The power to detect marker–trait associations (MTA) in GWAS depends on the extent of genetic diversity within the panel, the accuracy and variability of phenotypic data, population structure, marker density, level of linkage disequilibrium, and experimental design that affect heritability (Lozada et al. 2022). Next-generation sequencing techniques such as genotyping-by-sequencing (GBS) further facilitate association mapping by generating millions of single nucleotide polymorphisms (SNPs), which could provide a comprehensive coverage of the genome for marker–trait associations (Lozada et al. 2021). Multilocus GWAS models such as mrMLM (Wang et al. 2016), ISIS EM-BLASSO (Tamba et al. 2017), pLARmEB (Zhang et al. 2017), FASTmrEMMA (Wen et al. 2018), pKWmEB (Ren et al. 2018), and FASTmrMLM (Tamba and Zhang 2018) are good alternatives to single locus because of their efficient computational approach, where markers are initially scanned and selected using a less stringent threshold of significance, after which, a likelihood test is implemented to confirm the true quantitative trait loci (Wang et al. 2016).

In recent years, high-throughput phenotyping (HTP) has emerged as a faster, smarter, accurate, and resource-efficient platform to measure horticultural traits under diverse field conditions for plant breeding, precision agriculture, and functional genomics (Yang et al. 2020). Multiple image-based HTP platforms, including visible light (RGB) imaging, thermal imaging, chlorophyll fluorescence, hyperspectral imaging, and tomographic imaging, have revolutionized fruit phenotyping (Abebe et al. 2023). These platforms are often integrated with user-friendly graphical user interface (GUI) software and data management tools for efficient data processing. Tomato Analyzer (TA) is a GUI software that employs 2-dimensional images for the collection of accurate and detailed morphological and colorimetric data for various vegetable fruits (Rodríguez et al. 2010). The TA was originally developed for tomato (*Solanum lycopersicum*) and was later used to characterize fruit architecture and morphology in different vegetables, including chile peppers (Nankar et al. 2020; Khokhar et al. 2024). The software can measure more than 30 fruit morphology attributes, organized into 10 categories, from two-dimensional images in a semiautomatic and reproducible manner. Among these 10 categories, basic fruit measurements capture overall fruit morphology, providing essential information for vegetable breeders (Rodríguez et al. 2010).

The integration of phenomics and genomics for MTA studies in vegetables is a rapidly evolving area aimed at enhancing crop improvement through precise trait mapping and breeding (Mansoor et al. 2025). Recent advancements in genomic databases, combined with improvements in HTP platforms, have facilitated the identification of novel loci and genes, thereby enhancing genetic gain even for traits with low heritability (Pratap et al. 2019). For the current study, we integrated genomics and phenomics to identify genomic regions associated with fruit morphology. The objectives were to: (i) characterize fruit phenotypic variability of chile peppers using TA, an image-based phenomics tool; (ii) implement multilocus GWAS models to identify marker–trait associations for fruit morphology and yield; and (iii) determine candidate genes regulating fruit morphology.

## Materials and methods

### Plant material and experimental design

A *Capsicum* association mapping population (CAMP), consisting of 129 genotypes from the New Mexico State University (NMSU) Chile Pepper Breeding and Genetics Program, was used to conduct MTA analyses for fruit architecture and morphology. Seeds for phenotyping and genotyping were initially sown in F-1020 multicell trays (American Horticultural Supply, Inc., California, United States) and grown under greenhouse conditions (Sharma et al. 2017) at the Fabian Garcia Science Center, NMSU, Las Cruces, New Mexico (32°16′46.7″N, 106°46′24.7″W). Seedlings with 8 to 10 true leaves were transplanted in an augmented block design with ∼25 cm (10 inches) apart in 4.5 m (15 ft.) plots with 1 m (∼3 ft.) between plots to maintain at least 15 plants per plot and per genotype at Leyendecker Plant Science Research Center, Las Cruces, New Mexico (32°11′58.1″N 106°44′30.5″W) for the 2024 growing season. The CAMP was evaluated in 10 blocks with an unequal number of genotypes (minimum 10 and maximum 15), with 3 checks [Charger (New Mexican pod type), Red Rocket (cayenne), and Spada (jalapeño)] replicated in each block. Augmented designs are commonly used in plant breeding field trials to evaluate large diversity panels, where unequal numbers of genotypes were tested with blocking effects against checks in an unreplicated fashion, saving time and resources without compromising the critical differences between the tested treatments (Federer et al. 2001).

### High-throughput phenotyping and phenotypic data analysis

**Fig. 1.**
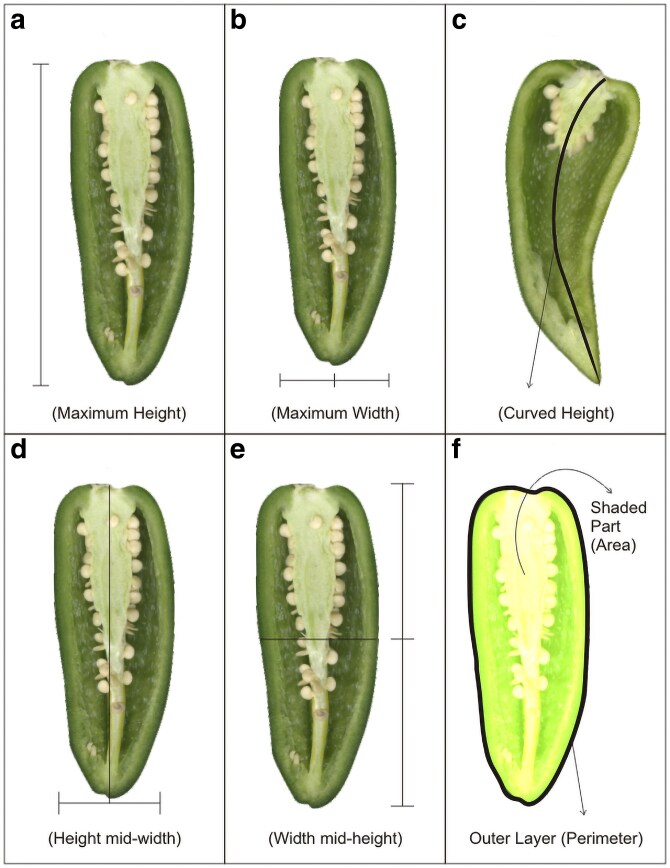
Illustration of fruit morphology descriptors: a) Maximum height (MAXH), b) Maximum width (MAXW), c) Curved height (CURH), d) Height mid-width (HMW), e) Width mid-height (WMH), f) Area (ARA), and perimeter (PER).

A total of 20 fruits, 10 each for red and green fruit samples, were collected randomly from five plants for each genotype. Mature fruit samples were taken between ∼150 and 180 d after transplanting. Fruit sample preparation, scanning, and image processing were conducted as described previously by Khokhar et al. (2022). Briefly, fruit samples were washed, cleaned, and blot-dried before cutting longitudinally through the center using a serrated knife. Fruit samples were scanned using an Epson Expression 12000XL Photo scanner (Epson, Inc., California, United States). Scanned images were processed for fruit morphology descriptors (Fig. 1), including maximum height (MAXH, cm), maximum width (MAXW, cm), width mid-height (WMH, cm), height mid-width (HMW, cm), curved height (CURH, cm), perimeter (PER, cm), and area (ARA, cm^2^) using the Tomato Analyzer v.4.0 software (Ramos et al. 2018). The weight of 10 fruits (5 mature green and 5 mature red), randomly selected from five plants of each genotype, was recorded as PODW. Yield was represented as the weight (in kg) of mature red and green pepper fruits harvested from five randomly selected plants per genotype. Analysis of variance was performed using the “auganova” function from the “augmentedRCBD” package (Aravind et al. 2021) as described by Federer (1961) to detect the mean differences for fruit morphology.

Descriptive statistics analysis was conducted to establish minimum values, maximum values, mean values, standard deviation, standard error, and coefficient of variation (CV). Pearson correlation coefficients between fruit morphology descriptors were computed using the “corr*_*coef” function from the “metan” package (Olivoto and Lúcio 2020). Best linear unbiased predictions (BLUPs) (Harville 1990) were estimated using a mixed model approach with the “asreml” package (Butler et al. 2017), where test genotypes and blocks were modeled as random effects and checks were treated as fixed effects. This approach reduced the environmental noise and provided a baseline for the comparison of test genotypes with checks. Genomic relationship matrix from “rrBLUP” package was calculated for additive variance (AV), dominance variance (DV), and residual variance (RV) using genotype-by-sequencing (GBS) data to estimate the narrow-sense heritability (*h*^2^) with the “mmer” function from the “sommer” package (Covarrubias-Pazaran 2016) in R version 4.1.2 (R Core Team 2021).

### DNA extraction and genotyping-by-sequencing (GBS)

Young seedlings (30 to 45 d old) from individual plants for each genotype were used for the collection of leaf tissue samples using 1.2 mL Qiagen collection microtubes (Qiagen, Maryland, United States). Isolation of DNA using ∼50 mg of leaf tissue using the Qiagen DNEasy 96 plant extraction kits was performed through the University of Minnesota Genomics Center DNA extraction facility (https://genomics.umn.edu/service/dna-extraction). DNA quantification was done using PicoGreen (Thermo Fisher Scientific, Massachusetts, United States), and samples were prepared for genotyping-by-sequencing (GBS) after performing normalization to 10 ng/μL. GBS was performed as previously described in Lozada et al. (2021). Briefly, a single enzyme digestion protocol was implemented (*ApeKI*, New England Biolabs, Inc., Massachusetts, United States), followed by enzyme incubation, ligation, purification, and amplification, and barcodes were added to the DNA fragments. DNA libraries were purified, quantified, and pooled using solid-phase reversible immobilization (SPRI) beads. Illumina NovaSeq 6000 sequencer (Illumina, California, United States) was used for single-end 1 × 100 sequencing.

FASTQ files were generated by demultiplexing using the Illumina “bcl2fastq” software (Illumina, San Diego, California, United States). Trimmomatic (Bolger et al. 2014) was used to remove the first 12 bases from the 3′ ends of the reads to exclude adaptors. FASTQ files were aligned to the Zunla-1 (*C. annuum* L.) reference genome (Qin et al. 2014) using the Burrows–Wheeler Aligner (Li and Durbin 2010). Variant call format (VCF) files were processed using VCF tools to remove minor allele frequency < 1%. The VCF file was converted into HapMap format using TASSEL v.5.2.95 (Bradbury et al. 2007) for downstream GWAS analysis.

### Genetic diversity, population structure, and analysis of linkage disequilibrium

The output from the genetic diversity analysis in TASSEL v.5.2.95 was processed and visualized for minor allele frequency, major allele frequency, expected heterozygosity (*H*_e_), and polymorphic information content (PIC) using the “tidyverse” (Wickham et al. 2019) package in R v4.1.2. An admixture model with a burn-in of 1,000 iterations and 1,000 Markov chain Monte Carlo (MCMC) replicates was implemented using STRUCTURE (Pritchard et al. 2000) to evaluate the genetic stratification of the pepper population. The PGDSpider software (Lischer and Excoffier 2012) was used to convert the VCF file into STRUCTURE format before analyzing the data with STRUCTURE. The number of clusters was set between 1 and 10 for the admixed model, and the optimal number of clusters that best represent the CAMP was determined using the Evanno method (Evanno et al. 2005) through the “summariseQ” function of the POPHELPER 2.3.1 package (Francis 2017) in R v4.1.2. Linkage disequilibrium (LD) analysis for 40,709 SNP markers was performed using a sliding window approach with a window size of 50 (0.05 kb) in TASSEL v.5.2.95. A nonlinear regression model was fitted to LD coefficients (*r*^2^) for pairwise intrachromosomal markers against the physical distance (in Mb) of genome-wide SNP markers to detect the intersection point between the regression curve and the critical value of *r*^2^ at which LD started to decay.

### Genome-wide association study and candidate gene analysis

Genome-wide association mapping was implemented to identify SNP markers associated with fruit morphology. The unmapped markers and those SNPs with minor allele frequency < 0.05 were excluded from the analyses. Data imputation for missing values was done using the LD-KNNi imputation function (Money et al. 2015) from TASSEL v.5.2.95 software. A total of 40,709 SNP markers were used for GWAS. The multilocus random-SNP-effect Mixed Linear Model (“mrMLM.GUI”) package (Zhang et al. 2020) in R Version 4.1.2 was used to identify marker–trait associations using BLUPs for seven TA fruit descriptors (MAXH, MAXW, WMH, HMW, CURH, PER, and ARA). Six multilocus GWAS models, namely, Multilocus random-SNP-effect mixed linear model (mrMLM) (Wang et al. 2016), Fast multilocus random-SNP-effect mixed linear model (FASTmrMLM) (Tamba and Zhang 2018), Fast multilocus random-SNP-effect efficient mixed model association (FASTmrEMMA) (Wen et al. 2018), Polygenic-background-control based least angle regression plus empirical Bayes (pLARmEB) (Zhang et al. 2017), and Iterative modified-sure independence screening expectation–maximization Bayesian least absolute shrinkage and selection operator (ISIS EM-BLASSO) (Tamba et al. 2017) were used for association analysis. GWAS models included a kinship matrix (*K*) and five principal components (PC) to account for the population structure and genetic diversity of the panel.

Multilocus GWAS methods first scan the entire genome with a relaxed criterion (typically *P* < 0.01) to select a subset of potentially associated markers, removing highly correlated neighboring SNPs and reducing the number of markers for downstream analysis (Wang et al. 2016). These candidate markers were then used to fit a multilocus mixed linear model to estimate the markers' effects using empirical Bayes estimation, which applies shrinkage. Empirical Bayes estimation shrunk the effect size toward zero if the signal was weak, whereas the strong signals were preserved (Wang et al. 2016). Because of the multilocus nature and the shrinkage in empirical Bayes estimation, the effective number of independent tests is greatly reduced. This makes the method more powerful at detecting true associations while controlling false positives in GWAS for vegetable crops, where replication is expensive and trait heritability can be moderate. A threshold of LOD > 3.0 was applied to identify associated markers, as recommended by simulation and empirical studies for balanced power and false positive control (Wang et al. 2016; Wen et al. 2018; Zhang et al. 2019).

Candidate gene analysis was performed using EnsemblPlants (Bolser et al. 2016). The annotation file for CM-334; (Genome assembly: ASM512225v2) downloaded from the EnsemblPlants website (https://plants.ensembl.org/index.html) was used to find candidate genes located within 150 kb upstream and downstream of a SNP marker. Candidate genes were annotated (https://ensembl.gramene.org/index.html and https://uud.ncbi.nlm.nih.gov/home/genes/) based on biological processes and molecular functions for TA fruit morphology descriptors. All codes used in this study are publicly available at https://github.com/EhtishamSK (see Data availability).

## Results

### Phenotypic variability for fruit morphology and yield

Analysis of variance (ANOVA) showed highly significant differences (*P* ≤ 0.01) among test genotypes, checks, and checks vs test genotypes, indicating the presence of phenotypic variability for fruit morphology and yield parameters for CAMP (Table 1). PER (perimeter in cm) was the most variable trait, followed by ARA (area in cm^2^), CURH (curved height in cm), MAXH (maximum height in cm), and HMW (height mid-width in cm). MAXW (maximum width) and WMH (width mid-height in cm) were the least variable traits. The coefficient of variation (CV) ranged between 9.77 (WMH) and 54.20 (PODW). The average BLUP values for MAXH, MAXW, WMH, HMW, CURH, PER, and ARA were 9.04, 3.00, 2.03, 8.14, 9.40, 23.41, and 16.74, respectively (Supplementary Table 1). The average BLUP was 1.57 for yield and 0.19 for PODW.

**Table 1.** Analysis of variance for fruit morphology in the *Capsicum* association mapping population.

Source	Block (ignoring genotypes)	Genotypes (eliminating blocks)	Check	Genotype vs check	Residuals
DF	9	131	2	129	40
MAXH	19.82^a^	12.68^a^	104.55^a^	11.25^a^	1.04
MAXW	1.13^a^	1.20^a^	11.76^a^	1.04^a^	0.26
WMH	0.32^a^	0.52^a^	7.81^a^	0.41^a^	0.04
HMW	14.47^a^	9.52^a^	86.10^a^	8.33^a^	1.59
CURH	21.13^a^	13.60^a^	111.55^a^	12.08^a^	0.91
PER	108.27^a^	79.23^a^	676.96^a^	69.96^a^	8.24
ARA	83.59^a^	83.27^a^	1252.48^a^	65.14^a^	9.80
PODW	0.02	0.03^b^	0.45^a^	0.02	0.01
YLD	2.55^a^	0.73	9.21^a^	0.60	0.54

^a^Significant at 0.01 probability level.

^b^Significant at 0.05 probability level.

Abbreviations: MAXH, maximum height; MAXW, maximum width; WMH, width mid-height; HMW, height mid-width; CURH, curved height; PER, perimeter; ARA, area; PODW, 10-pod weight; YLD, yield.

### Heritability and trait correlation for fruit morphology and yield traits

**Fig. 2.**
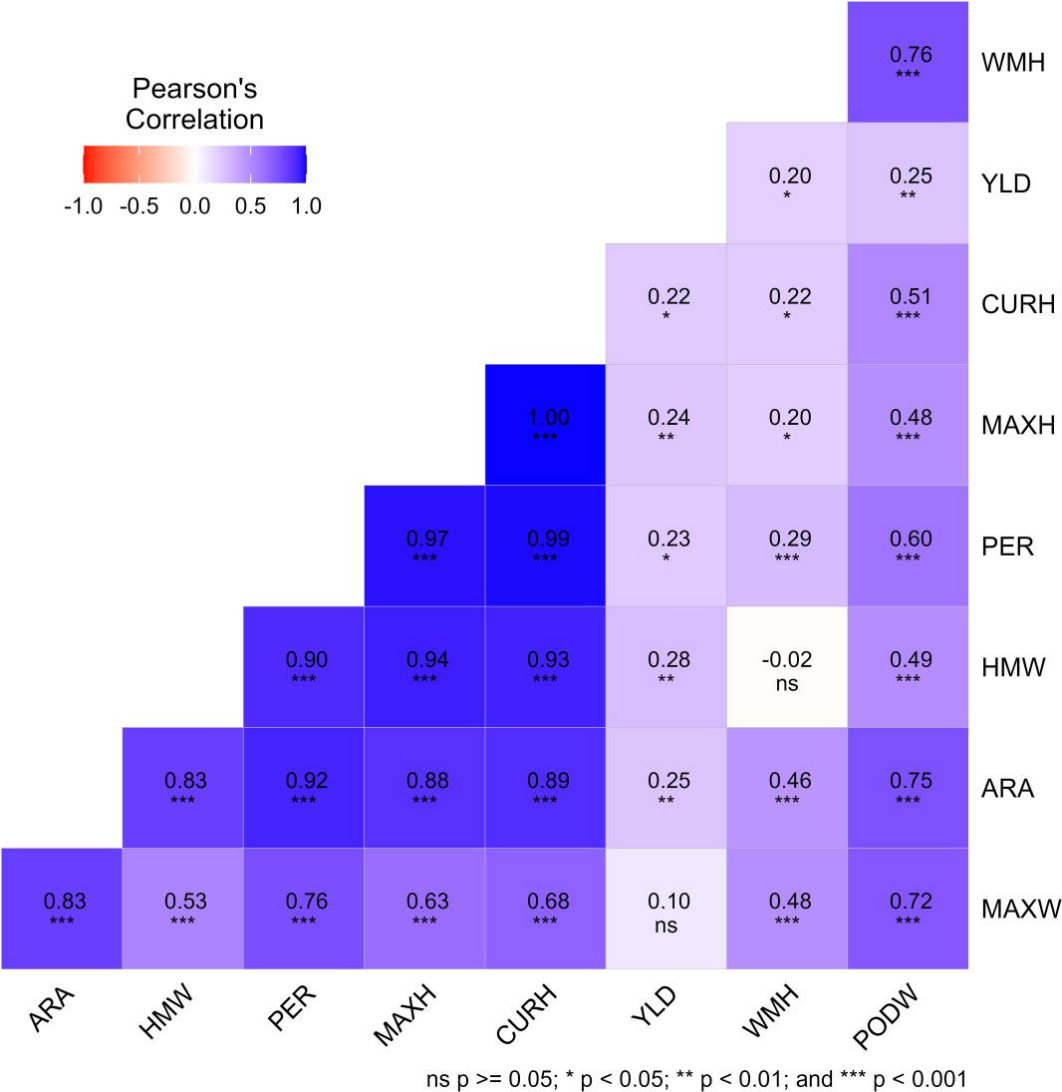
Heatmap showing Pearson correlation coefficients among nine chile pepper traits: area (ARA), height mid-width (HMW), perimeter (PER), maximum height (MAXH), curved height (CURH), yield (YLD), width mid-height (WMH), 10-pod weight (PODW), and maximum width (MAXW). Positive correlations were shown in shades of blue and negative correlations in shades of red, with color intensity indicating correlation strength. Strong positive correlations were observed among fruit size and shape traits, including ARA, PER, MAXH, CURH, WMH, and MAXW. Yield and 10-pod weight also showed positive associations with several fruit morphology traits. Asterisks indicated significance levels: *P < 0.05, **P < 0.01, and ***P < 0.001.

The total genetic variance was partitioned into additive variance (AV) and dominance variance (DV) to calculate narrow-sense heritability (*h*^2^). The *h*^2^ was greater than 0.30 for all fruit morphology traits (Table 2). The highest *h*^2^ was observed for WMH (0.33), followed by PODW (0.27), PER (0.26), and CURH (0.26), whereas the lowest *h*^2^ was observed for yield (0.18). Pearson correlation coefficient (*r*) ranged between 0.23 and 1.00. All traits were positively correlated; however, the strength of the correlation varied for different fruit morphology traits. WMH showed a weak correlation with MAXH (0.20), CURH (0.23), HMW (0.23), and PER (0.35). MAXW had moderate to high (>0.50 and <0.80) correlation with MAXH, CURH, HMW, PER, and ARA (Fig. 2). MAXW and CURH were highly correlated (>0.80) with ARA, PER, and HMW. YLD showed significant positive correlations (*r* > 0.20) with all fruit morphology traits.

**Table 2.** Summary statistics and genetic variability components for fruit morphology in *Capsicum* population.

Trait	Mean	Min	Max	SE	SD	CV	Additive genetic variance VA	Dominance genetic variance VD	Residual variance VR	Narrow-sense heritability *h*^2^
MAXH	9.00	2.07	15.47	0.29	3.33	10.95	1.084	1.619	1.837	0.24
MAXW	2.99	0.82	5.57	0.09	1.02	16.64	0.102	0.132	0.305	0.19
WMH	2.04	0.70	5.08	0.06	0.65	9.77	0.074	0.129	0.020	0.33
HMW	8.11	1.81	13.41	0.25	2.91	15.09	0.746	1.021	1.866	0.21
CURH	9.36	2.40	16.11	0.30	3.44	9.87	1.208	1.869	1.644	0.26
PER	23.32	6.48	38.80	0.72	8.24	11.93	7.063	11.130	8.462	0.26
ARA	16.72	0.77	32.58	0.71	8.14	18.10	6.722	10.117	11.087	0.24
PODW	0.20	0.10	1.01	0.01	0.16	54.20	0.008	0.010	0.028	0.17
YLD	1.54	0.66	4.82	0.08	0.90	46.02	0.002	0.003	0.002	0.28

Abbreviations: MAXH, maximum height; MAXW, maximum width; WMH, width mid-height; HMW, height mid-width; CURH, curved height; PER, perimeter; ARA, area; PODW; 10-pod weight; YLD, yield; Min, minimum; Max, maximum; SE, standard error; SD, standard deviation; CV, coefficient of variation.

### GBS-derived SNP markers

After filtration, imputation, and quality control, 40,709 GBS-derived SNP makers across 12 chromosomes were used for GWAS. The maximum number of SNP markers was reported for chromosomes (chr) 3 (5,319), followed by chr 6 (3,967), chr 2 (3,941), and chr 8 (3,658) (Supplementary Table 2). The least number of SNP markers was found for chr 11 (2,529). Marker density (SNP/Mb) ranges between 11.48 and 24.04. The highest SNP density (SNP/Mb) was exhibited by chr 2 (24.04), followed by chr 8 (23.86) and chr 3 (20.35), whereas the lowest SNP density was reported for chr 11 (11.48). Genome-wide markers were used to estimate the genetic diversity parameters, including MAF, *H*_e_, and PIC. The MAF ranged between 0.21 and 0.23, with a mean of 0.22. The reported MAF values represented the mean minor allele frequency per chromosome calculated across all SNPs. The average value for *H*_e_ was 0.30, with a range between 0.29 and 0.31. The average PIC was 0.26, with a maximum value of 0.27 (Supplementary Table 2).

### Genetic stratification and linkage disequilibrium

**Fig. 3.**
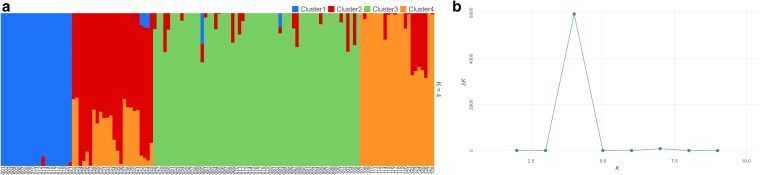
a) STRUCTURE plot illustrating population structure analysis of the CAMP at *K* = 4. The plot was composed of continuous colored regions representing four inferred genetic clusters across the genotypes. Cluster 1 is shown in blue, Cluster 2 in red, Cluster 3 in green, and Cluster 4 in orange. The *x*-axis represented the different CAMP genotypes. Variation in color proportions across the plot indicated differences in genetic background and admixture among genotypes. b) Delta K (*ΔK*) plot based on the Evanno method used to determine the optimal number of genetic clusters. A pronounced peak at *K* = 4 indicated that four clusters best explain the genetic structure of the CAMP.

The Evanno method revealed that *ΔK* = 4 represents the ideal number of clusters for CAMP (Fig. 3a and b). Cluster 1 consisted of 21 genotypes where the majority (17 genotypes) were breeding lines (Supplementary Table 1). A total of 24 genotypes were observed in Cluster 2, whereas 22 genotypes belonged to Cluster 4. All genotypes from both clusters were cultivars. Cluster 3 was the largest cluster with 61 genotypes. Unlike the other groups, Cluster 3 was genetically diverse and showed a strong admixture of cultivars, breeding lines, heirlooms, and landraces. While Clusters 1 and 4 were relatively uniform in pod type composition, Clusters 2 and 3 consisted of multiple pod types, including New Mexican, Banana, Bell, Cayenne, de Árbol, Habanero, Jalapeño, Paprika, Poblano, and Serrano (Supplementary Table 1).

**Fig. 4.**
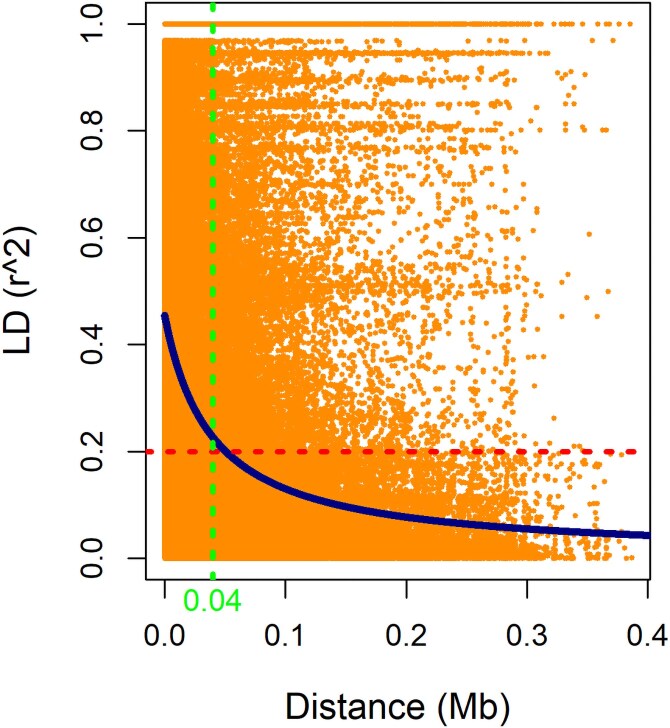
Linkage disequilibrium (LD) decay plot showing the relationship between pairwise genetic distance and LD (*r*^2^) values across the genome. The *x*-axis represented physical distance in megabases (Mb), and the *y*-axis represented LD measured as *r*^2^. Each data point represented the pairwise LD between two SNP markers plotted against their physical distance (Mb). A solid blue nonlinear regression curve illustrated the overall LD decay trend. A dotted red horizontal line marked the LD decay threshold at *r*^2^ = 0.20. A dotted green vertical line indicated the approximate genomic distance at which LD decays to half of its maximum value (~0.04).

A total of 313,205 intrachromosomal pairs were in significant LD (*P* < 0.05), whereas 682,241 pairs were in complete LD (*r*^2^ = 1.00) for the whole genome (Supplementary Table 2). Chr 3 had the largest number of intrachromosomal pairs in significant LD (40,697), followed by chr 6 (30,530). Likewise, chr 3 (85,802) and 6 (70,277) had the largest number of intrachromosomal pairs in complete LD (*r*^2^ = 1). The average distance for pairs in complete LD (1.89 Mb) was higher than that of pairs in significant LD (1.69 Mb). The average *r*^2^ value across the genome for significant pairs and pairs in complete LD was 0.08 and 0.17, respectively. Linkage disequilibrium (LD) decay analysis revealed a gradual decline in LD with increasing physical distance (Fig. 4). The nonlinear regression curve indicated that LD dropped to *r*^2^ = 0.20 at approximately ∼0.04 Mb.

### SNP markers associated with fruit morphology and yield

**Fig. 5.**
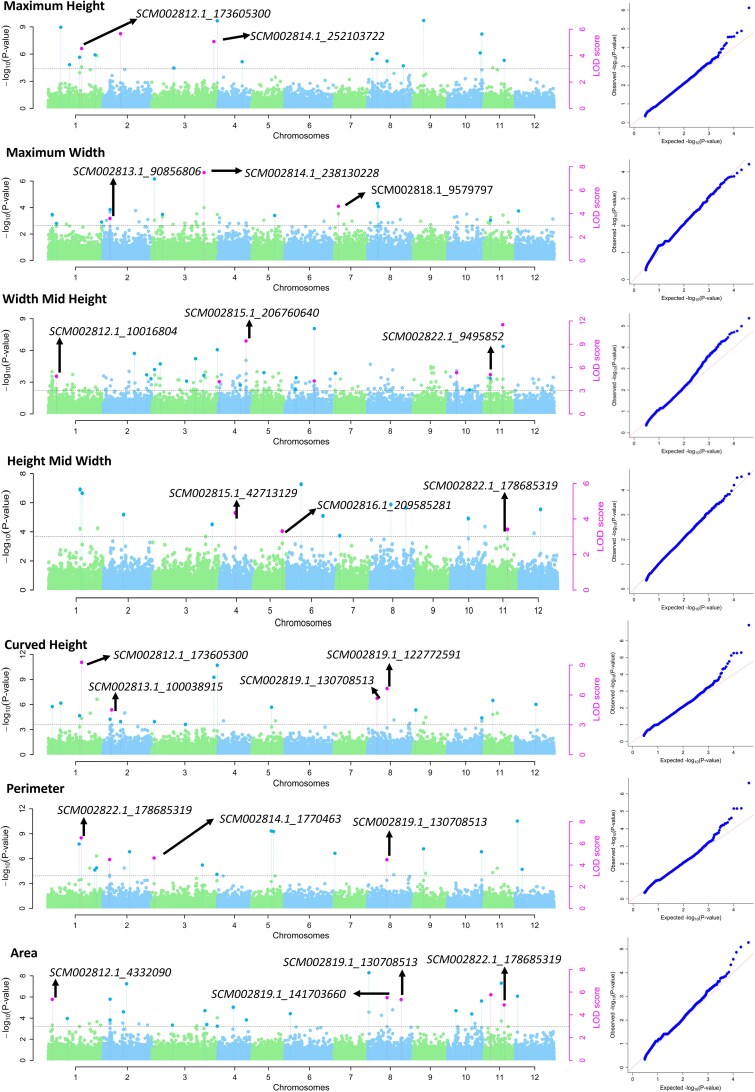
Manhattan plots and quantile–quantile (QQ) plots showing genome-wide association analysis results for fruit morphology traits. In the Manhattan plots, SNP markers were plotted across genomic positions on the *x*-axis, organized by chromosome, with −log10(*P*-value) on the *y*-axis. A horizontal threshold line indicated the LOD cutoff (>3.0), and peaks exceeding this threshold represented genomic regions showing association signals for fruit morphology traits. In the QQ plots, observed −log10(*P*-values) were plotted against expected values under the null hypothesis of no association. The diagonal line represented the expected distribution, and upward deviation from this line indicated departure from expectation and presence of association signals.

Multilocus GWAS models detected a total of 169 SNP markers associated with fruit morphology across the genome (Supplementary Table 3). The maximum number of SNP markers was detected for WMH (25), followed by PODW (23), ARA (23), CURH (18), MAXH (18), PER (17), TYL (17), HMW (14), and MAXW (14) (Fig. 5). There were 35 SNP markers found to be associated with fruit morphology and yield (LOD > 3.0) by at least two multilocus GWAS models, explaining 4.27% to 27.73% of the phenotypic variation (Table 3). Two SNP markers, *SCM002813.1_128055464* and *SCM002814.1_252103722*, detected by FASTmrMLM and FASTmrEMMA on chr 2 and 3 at 128.06 and 252.10 Mb, were associated with MAXH, and explained 7.00% to 9.49% of the phenotypic variation. Both SNP markers had a negative quantitative trait nucleotide (QTN) effect (−1.11 and −1.89) on MAXH (Table 3).

**Table 3.** SNP markers associated with fruit morphology in the Capsicum association mapping population.

Trait	SNP maker	Model(s)	Chr.	Marker position (Mb)	LOD score	*R* ^2^ (%)	Genotype	QTN effect
MAXH	*SCM002813.1_128055464*	FASTmrMLM, FASTmrEMMA	2	128.06	5.47	9.49	GG	−1.11
MAXH	*SCM002814.1_252103722*	FASTmrMLM, FASTmrEMMA	3	252.10	5.21	7.00	GG	−1.89
MAXW	*SCM002813.1_90856806*	ISIS EM-BLASSO	2	90.86	3.62	5.68	AA	0.25
MAXW	*SCM002814.1_238130228*	mrMLM, FASTmrMLM	3	238.13	8.26	19.94	GG	−0.42
MAXW	*SCM002818.1_9579797*	FASTmrMLM, FASTmrEMMA	7	9.58	5.29	7.22	TT	−0.69
WMH	*SCM002812.1_10016804*	FASTmrMLM, ISIS EM-BLASSO	1	10.02	5.42	4.36	AA	0.23
WMH	*SCM002815.1_1338681*	mrMLM, pLARmEB	4	1.34	7.48	7.24	AA	−0.22
WMH	*SCM002815.1_206760640*	mrMLM, FASTmrEMMA	4	206.76	14.35	27.73	GG	0.39
WMH	*SCM002817.1_73126596*	FASTmrEMMA, pLARmEB	6	73.13	4.26	7.59	CC	0.35
WMH	*SCM002821.1_17514441*	FASTmrMLM, pLARmEB	10	17.51	5.67	6.97	GG	−0.24
WMH	*SCM002822.1_9495852*	FASTmrEMMA, pLARmEB	11	9.50	6.14	12.12	AA	−0.70
WMH	*SCM002822.1_165467770*	mrMLM, FASTmrMLM	11	165.47	9.42	10.05	CC	−0.30
HMW	*SCM002815.1_42713129*	FASTmrEMMA, ISIS EM-BLASSO	4	42.71	4.51	4.66	AA	1.28
HMW	*SCM002816.1_209585281*	mrMLM, pLARmEB	5	209.59	3.07	11.68	GG	−1.07
CURH	*SCM002813.1_100038915*	FASTmrMLM, pLARmEB	2	100.04	4.81	4.29	AA	0.67
CURH	*SCM002819.1_122772591*	FASTmrMLM, pLARmEB	8	122.77	7.75	14.53	AC	−1.84
PER	*SCM002813.1_91245733*	mrMLM, FASTmrMLM, pLARmEB	2	91.25	5.67	4.27	TT	1.78
PER	*SCM002814.1_1770463*	FASTmrMLM, FASTmrEMMA, pLARmEB	3	1.77	6.01	8.45	TT	2.36
ARA	*SCM002812.1_4332090*	FASTmrMLM, FASTmrEMMA	1	4.33	8.21	7.95	TT	2.38
ARA	*SCM002819.1_141703660*	mrMLM, FASTmrMLM	8	141.70	6.30	6.35	TT	−1.84
ARA	*SCM002822.1_10111629*	mrMLM, pLARmEB	11	10.11	6.26	5.21	CC	1.67
PODW	SCM002815.1_41907871	FASTmrMLM, pLARmEB	4	41.91	5.73	14.43	CC	0.23
PODW	SCM002821.1_189438365	FASTmrMLM, FASTmrEMMA	10	189.44	16.34	16.07	CT	0.18
PODW	SCM002823.1_1900467	FASTmrMLM, pLARmEB	12	1.90	4.82	0.14	TT	0.14
PODW	SCM002814.1_253216001	ISIS EM-BLASSO, pLARmEB	3	253.22	3.09	0.48	TT	0.09
YLD	SCM002812.1_297277720	ISIS EM-BLASSO, FASTmrMLM	1	297.28	3.54	3.47	TC	0.21
YLD	SCM002813.1_36756187	ISIS EM-BLASSO, FASTmrMLM, pLARmEB, ISIS EM-BLASSO	2	36.76	5.18	21.22	CC	0.10
YLD	SCM002817.1_28144045	FASTmrMLM, pLARmEB, FASTmrMLM	6	28.14	10.47	23.56	AT	0.14
YLD	SCM002820.1_237750269	ISIS EM-BLASSO, FASTmrMLM	9	237.75	8.23	7.17	TA	0.47
YLD	SCM002821.1_190500764	ISIS EM-BLASSO, FASTmrMLM	10	190.50	4.60	4.55	AA	0.28
YLD	SCM002818.1_213106310	pLARmEB, ISIS EM-BLASSO	7	213.11	5.93	1.24	AA	0.32
YLD	SCM002820.1_238742688	pLARmEB, ISIS EM-BLASSO	9	238.74	4.78	1.84	AA	0.29
MAXH, HMW, CURH, PER	*SCM002812.1_173605300*	FASTmrMLM, pLARmEB, ISIS EM-BLASSO	1	173.61	10.67	8.90	CC	−1.61
HMW, ARA	*SCM002822.1_178685319*	FASTmrMLM, pLARmEB	11	178.69	3.47	4.93	GG	0.64
CURH, PER, ARA	*SCM002819.1_130708513*	FASTmrMLM, FASTmrMLM, pLARmEB	8	130.71	4.65	12.04	GG	2.65

Abbreviations: MAXH, maximum height; MAXW, maximum width; WMH, width mid-height; HMW, height mid-width; CURH, curved height; PER, perimeter; ARA, area; PODW, 10-pod weight; YLD, yield.

A SNP marker on chr 3 (*SCM002814.1_238130228*; LOD = 8.26) detected by two different multilocus GWAS models (mrMLM and FASTmrMLM) explained 19.94% of the phenotypic variation for MAXW (Table 3). Other SNP markers associated with MAXW were identified on chr 2 (*SCM002813.1_90856806*) and chr 7 (*SCM002818.1_9579797*), accounting for 5.68% to 7.22% of the phenotypic variation. Negative QTN effects were found for SNP markers associated with MAXH except for *SCM002813.1_90856806* (0.25). Another SNP marker (*SCM002815.1_206760640*; LOD = 14.35) detected by mrMLM and FASTmrEMMA models on chr 4 explained 27.73% of the phenotypic variation for WMH. Two SNP markers, *SCM002815.1_42713129* (*R*^2^ = 1.28) and *SCM002816.1_209585281* (*R*^2^ = 11.68) on chr 4 and 5, detected by two different multilocus GWAS models, were associated with HMW. Three SNP markers, *SCM002819.1_141703660*, *SCM002816.1_209585281*, and *SCM002819.1_122772591*, explained 6.35%, 11.68%, and 14.53% of the phenotypic variation for ARA, HMW, and CURH, respectively.

For yield parameters such as PODW and YLD, a total of 11 different SNP markers exceeded the LOD threshold across two models, exhibiting positive QTN effects. Among 35 SNP markers detected in more than two models, three were associated with multiple traits (Table 3). The SNP marker *SCM002812.1_173605300* (chr 1) was associated with MAXH, CURH, and PER. Similarly, *SCM002822.1_178685319* (chr 11) showed associations with HMW, PER, and ARA. The third multitrait marker, *SCM002819.1_130708513* (chr 8), was linked to CURH, PER, and ARA.

### Allelic variation for trait-associated loci

**Fig. 6.**
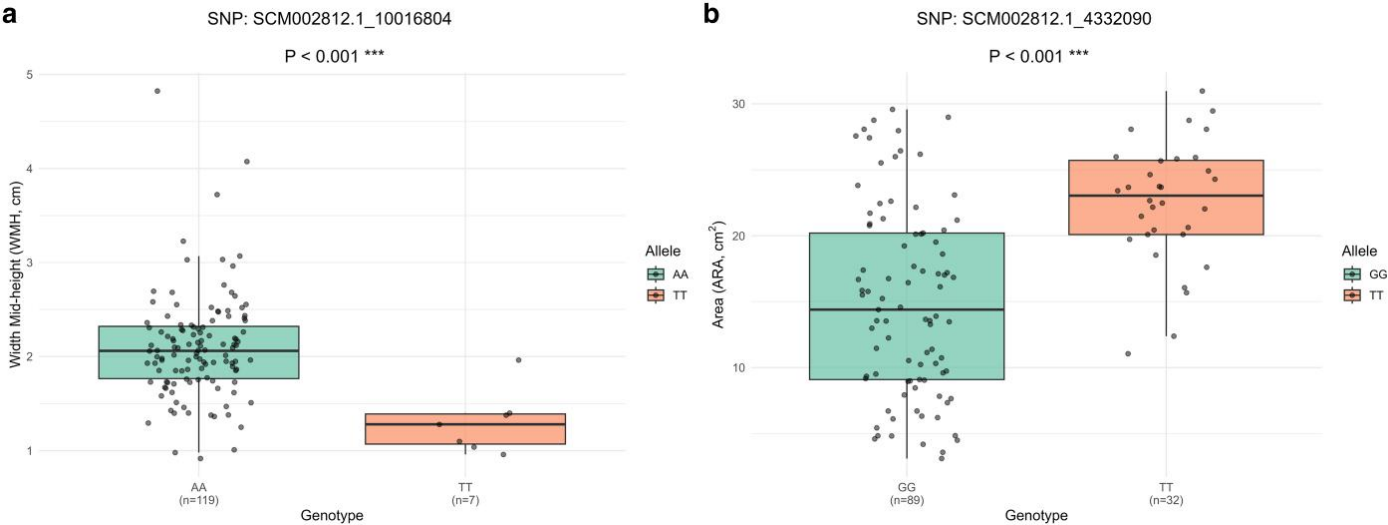
Allelic effects of two significant SNP markers associated with width mid-height (WMH) and Area (ARA). a) Box plots for SNP *SCM002812.1_10016804* (*P* < 0.001). The *y*-axis showed Width Mid-height (WMH, cm) ranging from 1 to 5 cm. Genotypes with AA allele (n = 119) had a median of 2.1 cm and TT (n = 7) had a median of 1.3 cm. The AA allele was associated with increased WMH. b) Box plots for SNP *SCM002812.1_4332090* (*P* < 0.001). The *y*-axis shows Area (ARA, cm^2^) ranging from 5 to 32 cm^2^. Genotypes are GG (n = 89) with a median of 14 cm^2^ and TT (n = 32) with a median of 23 cm^2^. The GG allele was associated with increased ARA.

Allelic variation for 12 SNP markers was observed to have effects on fruit morphology-related traits (Supplementary Fig. 1). Significant differences for the mean phenotypic values of ARA and HMW with contrasting alleles at two key SNP markers were noted (Fig. 6). The “AA” allele for *SCM002812.1_10016804* was associated with higher phenotypic values (*P* < 0.001) for WMH suggesting wider fruits. Similarly, for *SCM002812.1_4332090* individuals with the “GG” allele exhibited significantly higher values (*P* < 0.001) for ARA. Allelic variation for another SNP marker, *SCM002813.1_90856806*, was observed for MAXW.

Genotypes with allele “AA” showed a higher mean for MAXW, whereas those with allele “TT” were associated with a lower mean for MAXW. Similarly, the SNP marker *SCM002822.1_9495852* showed that genotypes with allele “AA” displayed a higher mean compared to genotypes carrying the “CC” allele for WMH. Allelic variation at SNP *SCM002822.1_178685319* showed that the allele “GG” was associated with higher phenotypic values for HMW, whereas those with “AA” allele corresponded to narrower fruit width (Supplementary Fig. 1). There was a substantial difference in phenotypes for allelic variation at *SCM002813.1_100038915* associated with CURH. Individuals possessing the allele “AA” showed longer fruits, whereas those genotypes carrying allele “GG” had shorter fruits. Significant difference among the phenotypes was apparent for *SCM002812.1_4332090* associated with ARA. The “GG” allele corresponded to a larger fruit area, whereas the “TT” allele was linked to a smaller fruit area.

### Candidate genes for fruit morphology

Candidate gene analysis was conducted for SNP markers identified in at least two multilocus GWAS models for various fruit morphology traits (Supplementary Table 4). A total of 15 candidate genes were found across 10 loci exceeding the LOD threshold. Among these, *YABBY4*, *FRIGIDA*-like protein 3, and *COBRA*-like protein were biologically relevant and functionally informative genes for fruit-related traits **(**Table 4**)**. Candidate genes with indirect roles in regulating fruit morphology included putative methyltransferase *PMT5* (methylation), auxin-responsive protein *SAUR65* (plant growth hormones), and glutamate–cysteine ligase (flowering time). Other candidate genes showed no direct functional relevance to fruit development pathways. Subsequent biological interpretation was focused primarily on *YABBY4*, FRIGIDA-like protein 3, and COBRA-like protein as the key candidate genes underlying the GWAS signals for fruit morphology traits.

**Table 4.** Candidate gene analysis identified potential genes regulating fruit morphology.

Gene Function	Candidate gene(s)	Trait(s)
Fruit and flower development	*YABBY4, FRIGIDA-like protein 3*, *COBRA-like protein*, *glutamate–cysteine ligase*	ARA, WMH, PODW, CURH
Cell division	*Cyclin-B2-4*	CURH
Plant growth hormones	*Auxin-responsive protein SAUR65*	PODW
Methylation	*Putative methyltransferase PMT5*	PODW

Abbreviations: WMH, width mid-height; ARA, area; CURH, curved height; PODW, 10-pod weight.

## Discussion

Dissecting the genetic architecture of fruit morphology could provide valuable information for developing pepper varieties that could meet consumer and grower needs and support mechanization. Multiomics approaches could enhance precision in identifying genomic regions associated with various horticultural and agronomic traits in *Capsicum* (Lozada et al. 2022). For this study, we integrated GWAS with a high-throughput phenotyping tool, Tomato Analyzer, to identify marker–trait associations for fruit morphology and yield-related traits in pepper. Trait correlations, variance components, and narrow-sense heritability were evaluated to develop a better understanding of the relationships between fruit morphology traits. The effects of allelic variation for associated SNP loci for fruit morphology traits were investigated. Subsequently, candidate genes within the proximity of 150 kb of the SNP markers exceeding the LOD threshold were identified for their potential roles in regulating fruit development.

### Genetic diversity in the *Capsicum* population is associated with phenotypic variation in fruit morphology traits

The CAMP displayed significant phenotypic diversity for fruit morphology traits. Moderate to low narrow-sense heritability estimates (*h*^2^) indicated that the additive genetic variance had a modest contribution toward the expression of the phenotype, thereby making response to selection slow due to significant nonadditive or environmental effects (Bernardo 2002). The pattern implies that fruit morphology is regulated by a complex genetic architecture, where nonadditive genetic effects and environmental factors play a significant role in phenotypic expression. A strong, positive, and highly significant Pearson correlation between the fruit morphology traits was consistent with correlation coefficients reported in previous studies (Joukhadar et al. 2024; Khokhar et al. 2025); however, all fruit morphology traits showed moderate to weak positive correlation with yield. Nevertheless, the positive correlation of all fruit architecture traits with pod weight indicated that selection for improved fruit shape may enhance yield potential (Khokhar et al. 2025).

Analysis using STRUCTURE and Neighbor joining tree revealed two distinct genetic clusters within the CAMP, indicating the presence of subpopulations, despite the panel being composed exclusively of *C. annuum*. This intraspecific genetic stratification may be associated with the considerable fruit phenotypic diversity present in the CAMP, which included 11 distinct pod types. The observed variation in fruit shape is likely due to selection for various pod types, reflecting diverse breeding goals and market preferences within the *C. annuum*. Kaur et al. (2024) previously reported seven distinct clusters, whereas Khokhar et al. (2024) identified two subpopulations in diverse *C. annuum* populations. The rate at which LD decay determines the number of markers needed for the association mapping (Abdurakhmonov and Abdukarimov 2008). Few markers are needed if there were a gradual decrease in LD (extensive LD); however, steep LD decay (rapid LD) comparatively requires more markers for higher genome resolution in marker–trait association analyses (Gupta et al. 2005). For the current study, a rapid decline in LD at ∼0.04 Mb was observed. This decline in LD may be partially explained by 11 distinct pod types in the CAMP associated with historical recombination events and selection that have contributed to the genetic diversity within the population. Consistent with previous investigations, a rapid LD decay was reported at 0.13 Mb (Nimmakayala et al. 2016), 0.14 Mb (Lee et al. 2020), and 0.5 Mb (Wu et al. 2019) in *C. annuum*.

### Marker–trait associations for fruit morphology and yield reflect genetic complexity of the traits

Multilocus GWAS identified a total of 169 SNP markers across 12 chromosomes. Thirty-five markers exceeded the LOD threshold for at least two models. Our findings were consistent with previous studies, which reported multiple QTL regions associated with fruit length, fruit width, area, perimeter, and fruit shape on chr 1 to 12, indicating the complex genetic architecture of fruit morphology in chile pepper (Chaim et al. 2001; Yarnes et al. 2013; Hill et al. 2017; Chunthawodtiporn et al. 2018; Lee et al. 2020; Ro et al. 2024). Chr 1, 2, and 3 showed the highest number of marker–trait associations for fruit morphology.

A total of 50 marker–trait associations were identified for fruit length including MAXH, CURH, and HMW. Many of these loci colocalized with previously reported genomic regions, although the exact positional concordance was limited (Table 5). For example, a locus on chr 4 identified to be previously associated with fruit length at 211.85 Mb (Lee et al. 2020) was flanked by a SNP identified 10.28 Mb upstream in this study, suggesting additional GWAS signals within the same chromosome. Similarly, SNP markers detected on chr 1, 8, and 10 for MAXH were located within a few megabases of previously reported regions (Solomon et al. 2019), indicating partial overlap but not exact correspondence. Some overlaps with biparental QTL studies were also observed. Notably, two markers on chr 1 were reported within the *FT1.1* interval for MAXH (Chunthawodtiporn et al. 2018). Additional associations for MAXH and CURH were located in proximity to previously reported QTLs across chr 1 to 3, with several markers reported within or near QTL intervals reinforcing the relevance of these genomic regions for fruit morphology (Rao et al. 2003; Yarnes et al. 2013; Han et al. 2016). For MAXW and WMH, several SNP markers were mapped near previously reported loci. On chr 2, two markers were located within 1.5 to 6.0 Mb downstream of the *FWI2.1* region (Lopez-Moreno et al. 2023). Additional overlaps for MAXW and WMH were observed with loci reported in diverse germplasm panels further supporting the importance of these regions (McLeod et al. 2023; Ro et al. 2024). The SNP markers for fruit perimeter and area showed stronger positional consistency with prior studies. Several markers on chr 2 and 3 were located within reported QTL intervals (Yarnes et al. 2013; Chunthawodtiporn et al. 2018), indicating stable genomic regions controlling these traits across populations. Overall, the majority of the associations identified in this study were localized to genomic regions previously mapped for fruit morphology and yield but with positional shifts on a few to several megabases. These discrepancies likely reflect differences in population structure, marker density, and LD, and highlight the complex genetic architecture of fruit morphology in chile pepper.

**Table 5.** Positional comparison of marker–trait associations with previously reported QTLs and SNP loci for fruit morphology in chile pepper.

Trait(s)	Chr	SNP	SNP position (Mb)	Reference locus (Mb)	Distance from reference locus (Mb)	Relative position	Reference
MAXH	4	*SCM002815.1_201613300*	201.61	211.85 (SNP)	10.28	Upstream	Lee et al. (2020)
MAXH	1	*SCM002812.1_17040477*	17.04	14.77 (SNP)	2.27	Downstream	Solomon et al. (2019)
MAXH	8	*SCM002819.1_130708513*	130.71	148.78 (SNP)	3.71	Upstream	Solomon et al. (2019)
MAXH	10	*SCM002821.1_147370253*	147.37	148.78 (SNP)	1.41	Upstream	Solomon et al. (2019)
MAXH CURH HMW	1	*SCM002812.1_166806748*	166.81	158 to 219 (QTL; *FT1.1*)	0^a^	Within interval	Chunthawodtiporn et al. (2018)
MAXH CURH HMW	1	*SCM002812.1_173605300*	173.61	158 to 219 (QTL; *FT1.1*)	0^a^	Within interval	Chunthawodtiporn et al. (2018)
MAXH CURH	1	*SCM002812.1_4332090*	4.33	13.8 to 19.6 (QTL; *1.1*)	0^a^	Within interval	Yarnes et al. (2013)
MAXH CURH	1	*SCM002812.1_17040477*	17.04	18.53 to 27.06 (QTL; *fl1.2*)	0^a^	Within interval	Rao et al. (2003)
MAXH CURH	3	*SCM002814.1_252103722*	252.10	240 to 252 (QTL; *FL3.5*)	0^a^	Within interval	Han et al. (2016)
MAXH CURH	3	*SCM002814.1_260220639*	260.22	240 to 252 (QTL; *FL3.5*)	0^a^	Within interval	Han et al. (2016)
HMW	3	*SCM002814.1_248991392*		240 to 252 (QTL; *FL3.5*)	0^a^	Within interval	Han et al. (2016)
WMH	2	*SCM002813.1_145137233*	145.14	155.7 to 157.7 (QTL; *FWI2.1*)	0^a^	Within interval	Lopez-Moreno et al. (2023)
WMH	2	*SCM002813.1_159418100*	159.42	155.7 to 157.7 (QTL; *FWI2.1*)	1.72	Downstream	Lopez-Moreno et al. (2023)
WMH	2	*SCM002813.1_163595456*	163.60	155.7 to 157.7 (QTL; *FWI2.1*)	5.90	Downstream	Lopez-Moreno et al. (2023)
MAXW WMH	1	*SCM002812.1_10016804*	10.02	12.64 (SNP)	2.62	Upstream	McLeod et al. (2023)
MAXW	3	*SCM002814.1_238130228*	238.13	234.16 (SNP)	3.97	Downstream	McLeod et al. (2023)
WMH	3	*SCM002814.1_260220639*	260.22	267.37(SNP)	7.15	Upstream	McLeod et al. (2023)
WMH	3	*SCM002814.1_260220639*	260.22	260.73 (SNP)	0.51	Upstream	Ro et al. (2024)
PER	2	*SCM002813.1_140030847*	140.03	138.16 to 168.71 (QTL; *XP2.1*)	0^a^	Within interval	Chunthawodtiporn et al. (2018)
ARA	2	*SCM002813.1_136571592*	136.57	138.16 to 168.71 (QTL; *XA2.1*)	0^a^	Within interval	Chunthawodtiporn et al. (2018)
ARA	3	*SCM002814.1_240923369*	240.92	240.84 to 244.36 (QTL; *3.1*)	0^a^	Within interval	Yarnes et al. (2013)
ARA	3	*SCM002814.1_244052666*	244.05	240.84 to 244.36 (QTL; 3.1)	0^a^	Within interval	Yarnes et al. (2013)

Reference locus represented either a reported QTL interval or SNP position from previous studies. For QTL intervals, distance was calculated as the minimum distance between the SNP and the nearest QTL boundary. For single SNP reports, distance represented the absolute difference in physical position. Relative position indicated whether the SNP was upstream or downstream of the reference locus. Abbreviations: MAXH, maximum height; CURH, curved height; HMW, height mid-width; WMH, width mid-height; PER, perimeter; ARA, area.

^a^Distances equal to 0 Mb indicated that the SNP was located within the reported QTL interval.

GWAS conducted using small population sizes could face challenges related to limited statistical power for identifying associations of modest effect size. While random chance could lead to false positives, small sample sizes may fail to detect true associations (Tam et al. 2019). Nonetheless, such studies could still provide valuable exploratory insights into the genetic architecture of complex traits. Small populations consisting of less than 100 genotypes have been used in GWAS for various traits in chile pepper (Nimmakayala et al. 2016; Khokhar et al. 2024), cucumber (*Cucumis sativus*; Kumar et al. 2022), and wheat (*Triticum aestivum*; Neumann et al. 2011). These small population sizes could still reveal associations when the traits are influenced by one or a few genes with large effects (Otyama et al. 2019). Consistent with these studies, the current study identified a limited number of SNP markers with large effects for fruit morphology using a population of 128 genotypes. GWAS successfully identified a SNP marker on chr 1 within the QTL region (*FT1.1*; Chunthawodtiporn et al. 2018) reported for fruit length (MAXH, CURH, and HMW) demonstrating the reliability of multilocus GWAS in relatively small populations with moderate heritability.

SNPs identified in the current study can facilitate the development of markers for major QTLs, enabling the fixation of favorable alleles for fruit size and shape in early breeding generations through marker-assisted selection (MAS). However, given the complex genetic architecture of fruit morphology traits, standalone MAS typically plateaus in capturing the full phenotypic variance and delivering sustained genetic progress. Complementing MAS with genomic selection, by training robust prediction models on genome-wide SNP data, would allow breeders to harness both major-effect alleles and the cumulative contribution of minor loci. This hybrid approach could accelerate the overall genetic gain and enhance selection efficiency across the breeding pipeline.

### Candidate genes associated with fruit architecture and morphology play important roles in plant development

Candidate genes with biological functions related to fruit, flower, and shoot development and flowering time were reported in the proximity of the SNP markers associated with fruit morphology. A candidate gene, *YABBY4*, was found near a SNP, *SCM002812.1_10016804* (chr 1), identified in multiple GWAS models. The *YABBY* gene family encodes plant-specific transcription factors that play an important role in flower, shoot, and leaf development, establishment of dorsoventral polarity, and response to abiotic stress (Zhang et al. 2019). The *YABBY* members are classified into five subfamilies, promoting lateral organ development in *Arabidopsis thaliana* (Rubio et al. 2002). Previously, tomatoes with an “extreme fruit size” phenotype were achieved due to a regulatory change of *YABBY*-like transcription factor resulting in 1,000 times larger fruit size than that of their wild relatives (Cong et al. 2008). *COBRA*-like proteins play a critical role in the biosynthesis of cell wall constituents that form the structural tissues of roots, stalks, leaves, and other vegetative organs (Cao et al. 2012). A homolog of the Arabidopsis *COBRA* gene, *SlCOBRA-like*, was identified in tomato (*S. lycopersicum*), and its functional role was established in fleshy fruit biology. The gene exhibited high expression during early fruit development; however, its expression declined during fruit ripening, indicating a primary role in early fruit developmental processes (Cao et al. 2012).

Another family of genes, the *FRIGIDA*-like *(FRL)*, present in all sequenced plant genomes, has roles beyond flowering, including embryonic development and seed maturation (Choi et al. 2009), found near a SNP (*SCM002817.1_10698796*; chr 6) associated with 10-pod weight. Seed maturation and embryo development are crucial for traits such as seed viability, germination rate, and fruit set, all of which are important in vegetable breeding programs. The *FRL* proteins primarily function as scaffold proteins, forming a protein complex with other regulators that can modulate the transcription of target genes (Vieira et al. 2019; Shen et al. 2022). Another candidate gene, glutamate–cysteine ligase regulates flower development by influencing flowering time through glutathione biosynthesis. The gene acts upstream of or within flower development, as mutations in the gene lead to delayed flowering due to reduced glutathione levels, indicating a regulatory role in the timing of the floral transition (Ogawa et al. 2001).

### Conclusion

Image-based phenomics and multilocus GWAS were employed to detect genomic regions associated with fruit morphology in chile peppers. A total of 169 SNP markers across 12 chromosomes were associated with basic fruit measurements derived from Tomato Analyzer. Among these, 35 SNPs exceeded the LOD threshold for at least two models. Chr 1, 2, and 3 showed the highest SNP marker coverage for fruit morphology traits. High trait correlation and medium narrow-sense heritability were observed. Candidate gene *YABBY4* is associated with plant development. The findings from the current study may be useful for chile pepper breeders in developing Kompetitive allele-specific markers (KASP) for marker-assisted selection and genomic selection for fruit morphology traits.

## Supplementary Material

jkag116_Supplementary_Data

## Data Availability

SNP marker information used in this study is publicly available on FigShare. Phenotypic data are available in Supplementary Table 1. All analyses were performed in R. Codes are publicly available at https://github.com/EhtishamSK. The repositories used for the analysis were as follows: https://github.com/EhtishamSK/AugRCBD.git (for augmented design); https://github.com/EhtishamSK/PopStr.git (for population structure); https://github.com/EhtishamSK/Single-Site-BLUPs.git (for single-site BLUPs); https://github.com/EhtishamSK/NS-Heritability.git (for narrow-sense heritability); https://github.com/EhtishamSK/LD-decay-plot.git (for linkage disequilibrium decay plot); and https://github.com/EhtishamSK/candigene.git (for candidate gene analysis). Supplemental material available at G3 online.
