# Supplementary figures and images for: Association mapping integrated with image-based phenomics identifies SNPs and candidate genes underpinning fruit morphology and development in chile pepper (Capsicum annum L.)
Source: G3 (Bethesda). 2026 May 2;16(7):jkag116. doi: 10.1093/g3journal/jkag116 (PMC13334168; doi:10.1093/g3journal/jkag116)

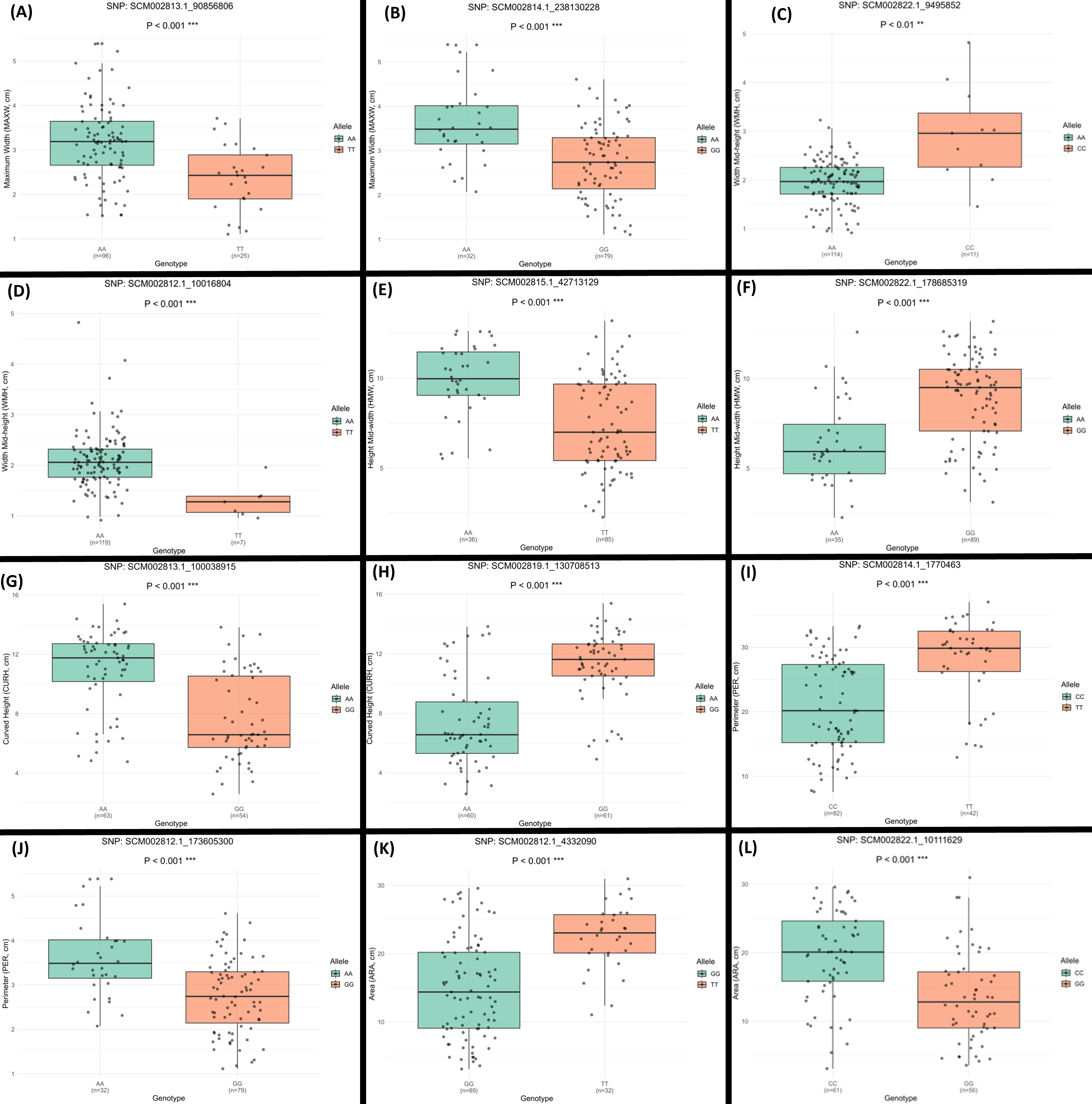

Supplement: jkag116_Supplementary_Data [file jkag116_supplementary_data.zip › Figure_S1_G3-2026-406773.png]
